# A Miniaturized Archimedean Screw Pump for High-Viscosity Fluid Pumping in Microfluidics

**DOI:** 10.3390/mi14071409

**Published:** 2023-07-12

**Authors:** Sinan Gucluer

**Affiliations:** Department of Mechanical Engineering, Aydin Adnan Menderes University, Aydin 09010, Turkey; sgucluer@adu.edu.tr

**Keywords:** microfluidic pump, micropump, high-viscosity fluid manipulation, lab-on-a-chip, fluid manipulation

## Abstract

Microfluidic devices have revolutionized the field of lab-on-a-chip by enabling precise manipulation of small fluid volumes for various biomedical applications. However, most existing microfluidic pumps struggle to handle high-viscosity fluids, limiting their applicability in certain areas that involve bioanalysis and on-chip sample processing. In this paper, the design and fabrication of a miniaturized Archimedean screw pump for pumping high-viscosity fluids within microfluidic channels are presented. The pump was 3D-printed and operated vertically, allowing for continuous and directional fluid pumping. The pump’s capabilities were demonstrated by successfully pumping polyethylene glycol (PEG) solutions that are over 100 times more viscous than water using a basic mini-DC motor. Efficient fluid manipulation at low voltages was achieved by the pump, making it suitable for point-of-care and field applications. The flow rates of water were characterized, and the effect of different screw pitch lengths on the flow rate was investigated. Additionally, the pump’s capacity for pumping high-viscosity fluids was demonstrated by testing it with PEG solutions of increasing viscosity. The microfluidic pump’s simple fabrication and easy operation position it as a promising candidate for lab-on-a-chip applications involving high-viscosity fluids.

## 1. Introduction

Microfluidics has been instrumental in the emergence and advancement of the field of lab-on-a-chip, enabling precise and efficient manipulation of small fluid volumes for a wide range of biomedical applications [1,2,3]. Microfluidic devices are widely used in a variety of domains, ranging from drug development and diagnostics to tissue engineering and point-of-care testing [4,5,6]. Microfluidic pumps, which provide controlled fluid manipulation, play a critical role at the core of lab-on-a-chip applications [7,8]. These pumps are important in transporting samples, reagents, and analytes within microfluidic devices, allowing chemical reactions and analysis to take place. Due to the intrinsic features of many biofluids, the ability to handle high-viscosity fluids in microfluidic devices is critical [9]. Most biofluids, such as blood, mucus, and synovial fluid, have higher viscosities than water. Precise control over the flow of these biofluids within microchannels is important for accurate measurements, efficient mixing, and optimum analyte delivery [10]. However, most of the existing microfluidic pumps are not capable of handling high-viscosity fluids [8,11,12,13].

Existing microfluidic pumps can be generally grouped into two main categories, active and passive, based on their operation principles [14,15,16]. Passive microfluidic pumps, which provide a simple device operation, have emerged as a potential alternative for fluid transfer in microfluidic devices [17]. These pumps create flow by utilizing capillary forces, gravity, or other intrinsic fluid features, without the use of external power sources or sophisticated actuation systems [18]. However, there are several limits to passive microfluidic pumps. One of the most significant issues is their low pumping efficiency, particularly when working with high flow rates or viscous fluids. Furthermore, differences in channel shape, fluid characteristics, and ambient conditions can all impact the performance of passive pumps, making it difficult to produce constant and predictable flow rates [19,20]. Furthermore, passive microfluidic pumps have limited control over the flow rate and on-demand flow modulation. They cannot actively control flow rates or react to changing experimental settings. Because of this constraint, they cannot be used in dynamic fluidic systems that demand precise manipulation and real-time modifications of flow profiles.

Active fluid manipulation systems implement external forces to direct fluid flow and thus offer more flexibility over the flow rate control compared to passive pumps. Several types of active microfluidic pumps have been developed employing various external means including mechanical actuators and magnetic, electric, acoustic, and optical fields [21,22,23,24,25]. Mechanically actuated micropumps including various types of peristaltic devices have been used in different forms to achieve controlled fluid pumping on a small scale [26,27]. These devices generally feature a flexible poly(dimethylsiloxane) (PDMS) layer and directional flow resistance to obtain a net flow rate [28]. While peristaltic micropumps are relatively simpler to fabricate, their inherited working mechanisms result in a highly pulsatile flow characteristic [27,29,30]. Furthermore, the pumping performance of peristaltic pumps significantly drops as the viscosity of the fluid increases [31]. Magnetic-field-based pumps employ magnetic impellers, cilia, valves, or diaphragms to drive fluids on-chip [32,33,34]. For example, multi-row arrays of artificial magnetic cilia were implemented to drive water inside microchannels with a flow rate of 11 µL/min, which was reported to be higher than that of previously demonstrated artificial ciliary systems [32]. In another study, Chen et al. demonstrated an implantable magnetic-field-driven pump by utilizing a multi-layer fluidic device and soft polymeric membranes [35]. Even though they showed dynamically controllable flow rates, device fabrication and actuation were usually quite complex in these examples. Furthermore, in almost all magnetic micropumps, the manipulated fluid is water, and the flow rates are generally between 1 and 20 µL/min [33,34]. Similarly, optical- and electric-field-driven micropumps require fairly complex setups and are generally designed to pump low-viscosity fluids such as water [16,36,37]. 

Acoustofluidic pumps represent a distinct category of microfluidic fluid manipulation tools that employ actuation mechanisms driven by acoustic fields [13,38,39,40,41]. Acoustic fields have been implemented in various ways to achieve manipulation of various samples in microfluidics due to their favorable characteristics such as biocompatibility, scalability, and dexterity [42,43,44]. Acoustofluidic pumping devices employ acoustic streaming phenomena to achieve directional and controllable fluid manipulation [45]. While acoustofluidic pumps have been shown to have versatile flow manipulation capabilities, they are generally implemented in water or similar low-viscosity fluid pumping applications [38,39,46]. In addition, acoustofluidic pumps usually rely on bulky and expensive peripheral equipment such as function generators and power amplifiers. Overall, due to the limitations of existing microfluidic pumps in manipulating higher-viscosity fluids, and the complexity of the fabrication and actuation of these devices, there is a need for a simple and low-cost microfluidic pump that is capable of pumping high-viscosity fluids. 

In this work, a miniaturized Archimedean screw pump was fabricated and applied to the pumping of high-viscosity fluids within microfluidic channels. Archimedean screw pumps were invented and implemented more than two thousand years ago for transporting large amounts of water from a lower altitude to a higher level by applying a tilt angle to the screw [47]. In this work, a miniaturized version of the Archimedean screw was 3D-printed and employed vertically to achieve microfluidic pumping without significant pulsing. Polyethylene glycol (PEG) solutions that are over 100 times more viscous than water were pumped using this micropump at only 3 V, implementing a basic mini-DC motor. Its simple fabrication and easy operation make this pump a strong and dexterous candidate for many lab-on-a-chip applications, especially those involving high-viscosity fluids.

## 2. Materials and Methods

**Figure 1:**
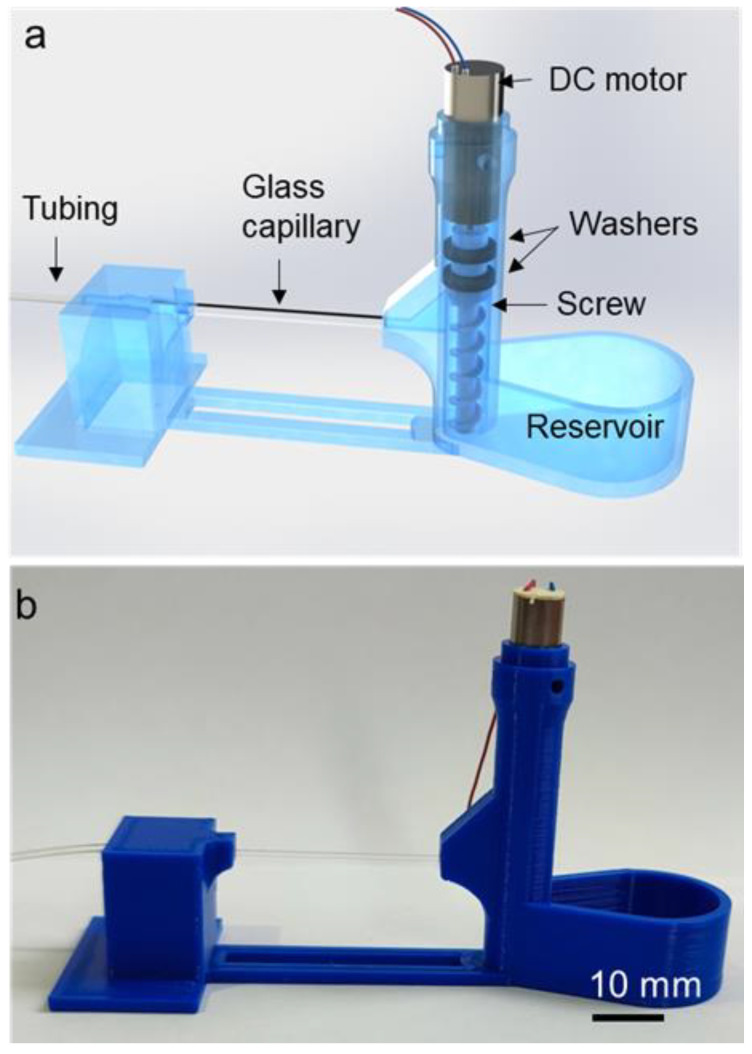
The screw pump: (**a**) schematic representation; (**b**) actual picture of the screw pump.

**Figure 2:**
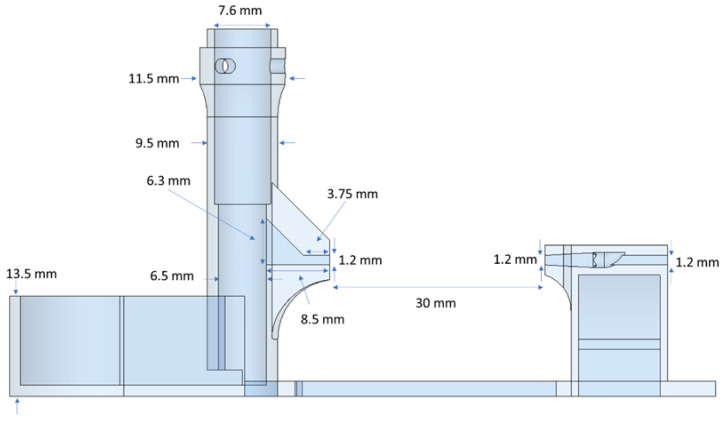
Detailed dimensions of the screw pump assembly.

**Figure 3:**
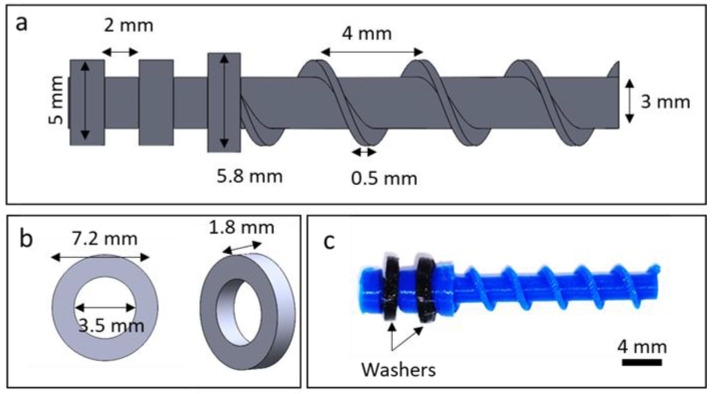
Dimensions and geometry of the screw: (**a**) schematic drawing of the screw; (**b**) washer dimensions; (**c**) actual picture of a screw with a pitch of 4 mm.

The screw pump was designed to have three main parts: screw, reservoir, and outlet adaptor with a glass capillary, as shown in Figure 1. In this design, the glass capillary was used to observe and characterize the fluid flow rate. The outlet adaptor was connected to the reservoir to ensure the stability of the whole assembly. The dimensions of the device are shown in Figure 2. The screw was devised to feature two indentations for washers and a small hole to be attached to a mini-DC motor. The pump was designed with reasonable minimum feature sizes that could be printed using a standard 3D printer. Three screws with pitch lengths of 2, 3, and 4 mm were designed. The dimensions of a screw with a pitch of 4 mm and a washer are shown in Figure 3. The washers were added to the screw design to prevent liquid from leaking from the top of the screw where the motor shaft was coupled to the screw. To achieve a tight fitting of the shaft of the motor, which has a diameter of 0.8 mm and a length of 6.2 mm, a hole with a 1 mm diameter and 6 mm depth was added to the top of the screw shaft. Considering that the dimensions of the printed parts can be slightly off, these dimensions were found to be adequate to prevent slipping of the motor shaft during rotation.

The reservoir and the screw were printed with a standard polylactic acid (PLA) filament using a fused deposition modeling (FDM) 3D printer, Ender 3 S1 (Creality 3D, Shenzhen, China). This particular 3D printer was chosen due to its direct extrusion capability that permits flexible filaments to be printed. Washers were printed using a polyurethane- and rubber-based TPU filament (Filameon, Kayseri, Turkey) with shore 90A hardness for flexibility and durability. The printed parts were heat-treated using a heat gun to obtain smooth surfaces. A square glass capillary (8260, VitroCom, Mountain Lakes, NJ, USA) with 0.6 × 0.6 mm inner dimensions and a 0.12 wall thickness was inserted through the outlet adaptor, and the perimeters of the adaptor ports were glued for a liquid-tight fitting using a minute epoxy (E340, Akfix, Istanbul, Turkey). Similarly, silicone tubing with a 0.5 mm inner diameter and 1 mm outer diameter was inserted through the outlet adaptor, and its perimeter was glued (Figure 1). To visualize the flow and characterize the flow rates, 5-micrometer-diameter polystyrene particles (Sigma Aldrich, Saint Louis, MO, USA) were added into water and PEG 700 (Sigma Aldrich, Saint Louis, MO, USA) solutions. The experiments were conducted using an optical microscope (SOIF BK500, Guangzhou, China), HD CMOS camera (HD-Ultra, Euromex, Arnhem, the Netherlands), and 1000 fps camera (Exilim EX-FC100, Casio, Tokyo, Japan). The flow rates were characterized using ImageJ software. For this, polystyrene particles were traced, and their average velocity values were calculated by dividing the distance by the time. Then, the velocity values were multiplied by the cross-sectional area of the glass capillary to obtain the volumetric flow rates. A small coreless DC motor (108990001, Seeed Studio, Shenzhen, China) was used to rotate the screw. An adjustable DC power supply (SPD-3606, GW-Instek, New Taipei City, Taiwan) was used to provide small increments in voltages.

## 3. Results and Discussion

**Figure 4:**
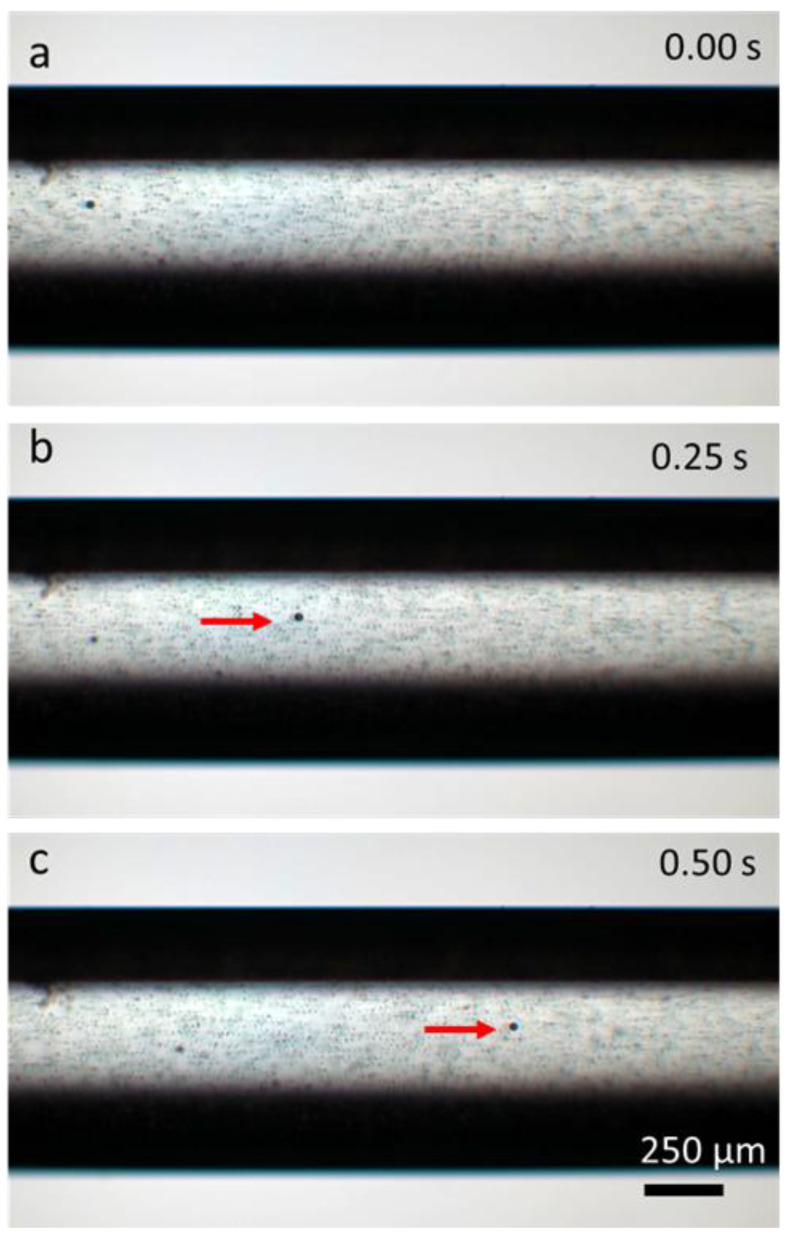
(**a**–**c**) The water–particle mixture is shown to be directionally manipulated at a 0.5 V applied voltage. The red arrow tracks a large particle indicating the flow direction.

The miniaturized Archimedean screw pump was assembled and tested using a water–polystyrene particle mixture, employing the screw with a 4 mm pitch length. Directional pumping of the water mixture is shown in Figure 4. It was observed that even at 0.5 V, directional and continuous fluid pumping could be generated. Compared to many of the existing micropumps, this is a very low voltage requirement that can be easily provided with a commonly available AA battery. This is a very important advantage that eliminates the dependence on peripheral bench-top equipment and enables point-of-care and field applications.

**Figure 5:**
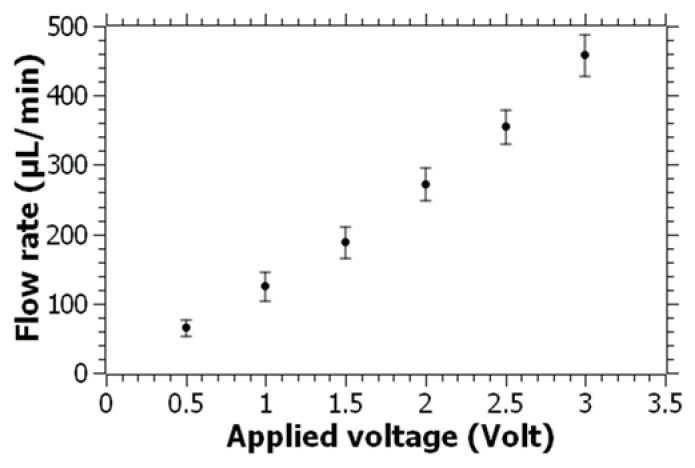
Measured flow rates for water as a function of the applied voltage. Error bars represent the standard deviation of 20 measurements for each voltage value.

**Figure 6:**
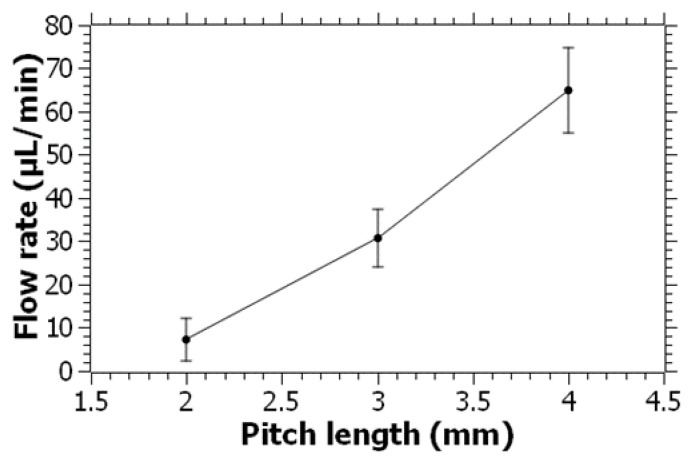
Flow rate characterization for water as a function of the pitch length of the screw at 0.5 V. Error bars represent the standard deviation of 20 measurements for each pitch length.

To better understand the fluid-pumping capability of the pump, the range of flow rates as a function of the applied voltage was characterized using water as the fluid. For this, the screw with a 4 mm pitch length was used, and the applied voltage was increased from 0.5 V to 3 V with 0.5 V increments. The range of flow rates as a function of the voltage is shown in Figure 5. The flow rate appeared to increase linearly with the applied voltage. At 3 V, flow rates of over 460 µL/min were obtained for water, which are well over the required flow rates for general-purpose lab-on-a-chip applications [48,49]. With the same pump configuration, an average flow rate of 64 µL/min was obtained at 0.5 V. For some applications involving chip cell or organoid experiments, a lower flow rate may be required. To lower the flow rate, we used screws with lower pitch lengths of 2 and 3 mm, which will have a smaller volume swept in each turn of the screw. Furthermore, as the pitch length decreases, the number of threads along the shaft of the screw will increase, which can slow down the screw due to the anticipated increase in the fluid resistance between the tight clearances of the screw thread and the housing. The flow rates for water at 0.5 V were characterized and plotted for the 2, 3, and 4 mm pitch lengths, as shown in Figure 6. It was observed that the flow rate dropped as the pitch length decreased. With a 2 mm pitch length, the flow rate dropped to less than 10 µL/min. As previously mentioned, the decrease in the pitch length increases the number of threads on the screw, which is likely to reduce the rotational speed of the screw at the same applied voltage. There are also other possible approaches that can be applied to reduce the flow rate by means of geometrical modifications. For example, the distance between the screw housing and the screw threads can be increased to let more pressure losses occur, which can potentially decrease the flow rate further at the same applied voltage. Alternatively, the geometry of the entrance of the glass capillary from the screw housing can be constricted to generate high flow resistance and decrease the flow rate passing through the capillary.

**Figure 7:**
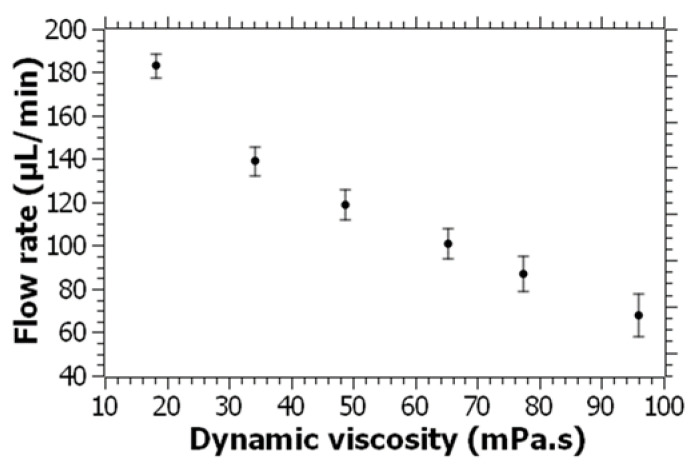
Pumping performance evaluation as a function of the viscosity at 3 V. PEG 700 and dilutions thereof were used as the fluids. Error bars represent the standard deviation of 20 measurements for each fluid.

The screw pump was tested with PEG solutions to demonstrate the system’s capacity to pump high-viscosity fluids. For this, PEG 700 dilutions of increasing viscosity were used, and the flow rates were characterized. Viscosity values between 18 and 96 mPa·s were used in the screw pump, and the applied voltage was maintained at 3 V for the experiments. It was found that even the highest-viscosity PEG 700 fluid, which is about 107 times more viscous than water, could be pumped at about 70 µL/min. A graph showing the flow rate versus the dynamic viscosity values is shown in Figure 7. Considering all the imperfections of the 3D-printed parts, and the higher tolerances between the parts, the performance of the pump is highly promising for handling fluids of even higher viscosity. Overall, the demonstrated pumping system is a very simple solution for pumping low- and high-viscosity fluids in microfluidic devices. Compared to the existing microfluidic fluid manipulation approaches, the presented device provides several benefits in terms of cost and performance. As for the cost, the presented device costs less than USD 4 including the DC motor, which is significantly lower compared to the cost of the majority of the existing micropumps considering the cost of cleanroom fabrication. In addition, powering this pump only requires only a few volts that can be provided through common batteries. When it comes to the performance of the system, both in terms of the maximum achievable flow rates for water and the viscosity of the fluids that can be handled, the presented pump is highly efficient and versatile. It is also important to discuss the power consumption of the pumping system for a better comparison and evaluation of the system. For the highest-viscosity PEG solution, at a 3 V applied voltage, 0.76 Amps of current is generated. In general, the power consumption of the system varied between 0.1 and 2.28 Watts based on applied voltages ranging from 0.5 V to 3 V. These values can be easily provided by common batteries, which further demonstrates that this miniaturized pump can be potentially used in field applications. 

Overall, the screw pump was demonstrated to be capable of manipulating fluids with a range of viscosities, including high-viscosity PEG solutions. The flow rate of the pumping device was also shown to be well regulated with the applied voltages. However, a better and more convenient mode of operation of the presented pump could be achieved by using a closed-loop control approach with a simple flow sensor. This way, the desired flow rates can be quickly achieved by precisely tuning the applied voltage to the DC motor. This can be especially useful considering the imperfections due to 3D printing and the higher tolerances that were designed for free rotation of the screw inside the housing. It is also important to discuss the fluid behavior within the pump when the pump is turned off or on. Due to the geometry and the principle of the screw pump, when the pump is turned off, the fluid starts to move back slowly due to gravity. As the viscosity of the fluid is increased, the backflow becomes smaller due to the increased flow resistance. As the pump is turned on, the flow almost immediately starts due to the very high rotational speeds that the DC motor provides. During the pumping, the flow is observed to be very stable, with no significant pulsing. 

While the demonstrated pumping system has many advantages such as higher flow rates and the ability to manipulate higher-viscosity fluids compared to the existing micropumps, it is also useful to compare this system to traditional syringe pumps. In this comparison, syringe pumps can be divided into two groups: commercial and 3D-printed syringe pumps. The commercial systems can provide very steady flow infusion, but they are fairly expensive and thus not accessible to many researchers in low-resource settings. Three-dimensionally printed syringe pumps have been demonstrated in conventional laboratory research and attracted significant attention due to their lower costs compared to their commercial counterparts [50,51,52]. Three-dimensionally printed syringe pumps provide a feasible and low-cost solution for fluid manipulation, but they still require stepper motors and electronic control units. The screw pump demonstrated in this study can still be considered a disposable device due to its smaller footprint and lower printing costs (under USD 2). Since fluids are in contact with the screw and the housing, the device can be replaced in each experiment thanks to the low cost of the entire assembly. Furthermore, syringe pumps, in general, are bulky and demanding for field applications due to their dependence on power outlets and relatively larger sizes. Finally, the presented screw pump can be constantly fed with liquids, or the reservoir can be designed to be much larger, whereas syringe pumps are restricted to the volume of the syringe.

Three-dimensionally printed parts can be prone to chemical corrosion depending on the type of filament used. In the demonstrated system, PLA was used as the main material, which is not resistant to organic solvents. For fluid manipulation experiments requiring organic solvents such as acetone or acidic or basic solutions, different filaments such as nylon or ABS should be used to increase the chemical resistance. Post-processing of the printed parts may also be required to prevent the release of plastic particles into the fluid. For the presented system, the PLA parts were heat-treated to obtain smooth surfaces and eliminate loose strings, which was sufficient to prevent any observable contamination of the fluids. Another approach involves the treatment of PLA surfaces with acetone vapor, which also results in smoother surfaces. Overall, it is important to consider the suitable types of materials for printing the screw pump based on the type of application. 

## 4. Conclusions

In conclusion, the field of microfluidics has significantly contributed to the development of lab-on-a-chip technologies, enabling precise manipulation of small fluid volumes for various biomedical applications. While microfluidic pumps are crucial components in these systems, most existing pumps are not capable of efficiently handling high-viscosity fluids. This limitation hinders their application in biomedical research and diagnostics, where accurate control over fluid flow is essential.

In this paper, a novel approach was presented by miniaturizing an Archimedean screw pump for microfluidic applications. The pump was fabricated using 3D printing technology, implementing a simple design consisting of a screw, reservoir, and outlet adaptor. The Archimedean screw pump was shown to be capable of efficiently pumping high-viscosity fluids within microfluidic channels. The experimental results showed that the screw pump achieved directional and continuous fluid pumping at a remarkably low voltage of 0.5 V, making it easily accessible and suitable for point-of-care and field applications. The flow rate of the pump is linearly proportional to the applied voltage, reaching over 460 µL/min at 3 V for water, which surpasses the requirements of many lab-on-a-chip applications. By using screws with smaller pitch lengths, the flow rate can be further reduced to meet specific experimental needs. Moreover, the screw pump was demonstrated to be capable of handling high-viscosity fluids by successfully pumping polyethylene glycol (PEG) solutions that are over 100 times more viscous than water. This feature makes the pump a promising tool for a wide range of lab-on-a-chip applications involving high-viscosity fluids.

This miniaturized Archimedean screw pump offers a simple and low-cost alternative for microfluidic pumping, addressing the limitations of existing pumps. Its ease of fabrication and operation, along with its ability to handle high-viscosity fluids, makes it a versatile and dexterous candidate for various biomedical applications. The development of such pumps opens up new opportunities for precise fluid manipulation, real-time modifications of flow profiles, and advances in lab-on-a-chip technologies.

## Data Availability

Data are available upon reasonable request from the corresponding author.
